# Efficacy, safety, and pharmacokinetics of MR13A11A, a generic of remifentanil, for pain management of Japanese patients in the intensive care unit: a double-blinded, fentanyl-controlled, randomized, non-inferiority phase 3 study

**DOI:** 10.1186/s40560-023-00698-9

**Published:** 2023-11-13

**Authors:** Matsuyuki Doi, Naoki Takahashi, Rumi Nojiri, Takehiko Hiraoka, Yusuke Kishimoto, Shinichi Inoue, Nobuyo Oya

**Affiliations:** 1 Department of Anesthesiology and Intensive Care Medicine, Hamamatsu University School of Medicine, 1-20-1 Handayama, Hamamatsu, Shizuoka 431-3192 Japan; 2 Clinical Development Department, Maruishi Pharmaceutical Co., Ltd., 2-2-18 Imazu-Naka, Tsurumi-Ku, Osaka, 538-0042 Japan

**Keywords:** Fentanyl, Intensive care unit, Mechanical ventilation, Pain management, Remifentanil

## Abstract

**Background:**

The aims of this study were to evaluate the efficacy, safety, and pharmacokinetics (PK) of continuous intravenous administration of remifentanil in mechanically ventilated patients in the intensive care unit (ICU).

**Methods:**

This was a multicenter, randomized, double-blinded, fentanyl-controlled, non-inferiority phase 3 study. Patients aged ≥ 20 years requiring 6 h to 10 days mechanical ventilation in an ICU and requiring pain relief were randomly assigned in a 1:1 ratio to receive either remifentanil (n = 98) or fentanyl (n = 98). Dose was titrated from an infusion rate of 1 mL/h (remifentanil: 0.025 µg/kg/min, fentanyl: 0.1 µg/kg/h) until the target level of analgesia (behavioral pain scale [BPS] ≤ 5 or numerical rating score [NRS] ≤ 3) was achieved by escalating the dose in 1 mL/h increasing. Administration was then adjusted to maintain the target level of analgesia until weaning from the ventilator. The primary endpoint was the proportion of patients who did not require rescue fentanyl. Safety was assessed according to standard procedures. PK of remifentanil in the arterial blood was assessed in 24 patients.

**Results:**

The proportion of patients achieving the primary endpoint in the remifentanil and fentanyl groups was 100% (92/92) and 97.8% (88/90), respectively. The difference between the groups was 2.2% (95% confidence interval, − 0.8–5.3) and non-inferiority of remifentanil to fentanyl was verified (*p* < 0.0001). The incidences of any adverse events in the remifentanil and fentanyl groups was 34 of 92 patients (37.0%) and 34 of 90 patients (37.8%), respectively. Adverse drug reactions was 12 in 92 patients (13.0%) and 15 in 90 patients (16.7%), respectively. In the PK analysis, blood remifentanil concentration decreased within 10 min to almost 50% of the end of administration, suggesting rapid offset of action following discontinuation of remifentanil.

**Conclusions:**

Remifentanil can be used safely for pain management in mechanically ventilated Japanese patients in the ICU.

*Trial registration*: Japan Registry of Clinical Trials, jRCT2080224954. Registered 20 November 2019, https://jrct.niph.go.jp/latest-detail/jRCT2080224954.

**Supplementary Information:**

The online version contains supplementary material available at 10.1186/s40560-023-00698-9.

## Background

MR13A11A (Remifentanil for intravenous injection “Daiichi Sankyo”®) was approved in Japan in 2016 as a generic of remifentanil and its indications were analgesia during induction and maintenance of general anesthesia for adults and maintenance of general anesthesia for pediatric patients [1]. Remifentanil is a potent, selective synthetic μ-opioid receptor agonist with an ultra-short–action [2]. It is rapidly metabolized by nonspecific plasma and tissue esterases to an inactive metabolite and is consequently unaffected by renal or liver function [3]. Onset of action of remifentanil is about 1 min, which quickly achieves steady state and the elimination half-life is very short with a context-sensitive half-life of 3–4 min, which is independent of the duration of infusion [4, 5]. The rapid onset and offset of a strong analgesic effect is highly predictable due to its characteristic pharmacokinetic profiles, without the risks of drug accumulation and effect prolongation observed with fentanyl and morphine [6]. These characteristics of remifentanil make it a useful agent for mechanically ventilated patients in the intensive care unit (ICU) setting, as patients commonly have some degree of organ dysfunction [4].

Remifentanil has been approved in over 80 countries, and is used for analgesia during general anesthesia, continuing analgesia until entering the ICU or postoperative care unit (PACU) after general anesthesia, and analgesia in the ICU. However, in Japan, remifentanil had not been approved for use in the mechanically ventilated patients in the ICU, and fentanyl and morphine were used for these patients [7]. Favorable clinical benefits of remifentanil in intensive care were demonstrated in several clinical studies [8–12]. Clinical practice guidelines for the management of pain recommend the use of remifentanil in the ICU as well as fentanyl and morphine [13–15].

Recent approval in Japan allows remifentanil to now be used for analgesia in the ICU, based on the results of clinical trials involving patients undergoing intensive care treatment, including mechanical ventilation following elective surgery or during medical management. Here, we report a phase 3 clinical trial to evaluate the efficacy and safety of continuous intravenous administration of remifentanil in mechanically ventilated Japanese patients in intensive care. We also report the pharmacokinetics (PK).

## Methods

### Study design and participants

This was a multicenter, randomized, double-blinded, parallel-group, fentanyl-controlled non-inferiority phase 3 study in patients requiring respiratory management in intensive care to evaluate the efficacy, safety, and pharmacokinetics of continuous intravenous administration of remifentanil (using MR13A11A as the investigational drug). The study was conducted at 29 sites in Japan from December 2019 to December 2020 (Additional file 1: Table S1).

The study protocol and the informed consent form were approved by the Ethics Review Board of Hamamatsu University School of Medicine, Hamamatsu, Japan, (Approval No: 709) and at each hospital listed in Additional file 1: Table S1. All patients gave written informed consent before initiation of any study-specific procedures. The study was conducted in accordance with the ethical principles originating in or derived from the Declaration of Helsinki and Good Clinical Practice guidelines. The study was designed and conducted by the sponsor in collaboration with the principal investigators. Maruishi Pharmaceutical Co., Ltd. monitored study conduct, collected the data, and performed the statistical analyses.

Inclusion criteria were male or female patients aged ≥ 20 years who required respiratory management due to intubation or tracheostomy for 6 h to 10 days in intensive care and who were anticipated to require pain relief. The patients entering the ICU after surgery were required to have an American Society of Anesthesiologist Physical Status (ASA) status of I–III at the pre-operative assessment. If a patient was a female of childbearing potential, she was excluded if pregnant, possibly pregnant, or lactating. Approximately 15% of the patients who were recruited from ICU had internal medicine as much as possible. Key exclusion criteria were patients with severe damage to the central nervous system, with a neurological disease that made pain/sedation assessment difficult as adjusted by the investigator, who had received nalmefene within one week prior to the study drug administration, who required local anesthetic, epidural or intrathecal administration of analgesics, or nerve block, those with contraindications to muscle relaxants, who were likely to suffer from respiratory depression such as coma due to head injury or brain tumor, those with a history of seizure or asthma, and patients who were assessed as suffering from severe illness, that might cause death within 24 h, and those with massive bleeding who were being considered for reoperation.

### Procedure

Patients who met the eligibility criteria were randomly assigned in a 1:1 ratio to receive either remifentanil or fentanyl. Treatment allocation of patients was initiated via an envelope method. Remifentanil and fentanyl can be distinguished by the appearance of their vial; therefore, to ensure masking was maintained for the patients, investigators, site staff, assessors, and sponsor, only unblinded persons appointed by the investigator prepared the blinded administration solution. The results of PK measurement were not reported to the investigators and sponsor before the primary analysis.

After entering ICU, pre-dose screening was performed under light sedation using sedatives as needed to maintain a Richmond Agitation-Sedation Scale (RASS) [16, 17] of -2 to 0. As a run-in treatment, the remifentanil group received placebo (physiological saline), and the fentanyl group received 1 to 2 μg/kg of fentanyl by slow intravenous bolus injection. The study drug was administered by continuous intravenous infusion during respiratory management under intubation or tracheostomy in the ICU. The duration of administration of the study drug was ≥ 6 h to ≤ 10 days. Dose titration was started from an infusion rate of 1 mL/h (remifentanil: 0.025 µg/kg /min in the remifentanil group, fentanyl: 0.1 µg/kg/h in the fentanyl group), and then escalated by 1 mL/h (remifentanil: 0.025 µg /kg/min, fentanyl: 0.1 µg/kg/h) until the target level [behavioral pain scale (BPS) [16, 18] ≤ 5 or numerical rating scale (NRS) ≤ 3] was achieved. After an effective infusion rate was obtained, the rate was adjusted to maintain the target analgesia level. If the target analgesic level was expected to be achieved without hindrance, further increases were continued. If the appropriate analgesic level was not obtained even after reaching the maximum infusion rate, fentanyl was administered as a rescue analgesic (open-label setting). If the target analgesic level was not achieved even after increasing the infusion rate four times in a row during 20 to 30 min in the maintenance phase, rescue use of fentanyl was to be considered. The maximum infusion rate was 20 mL/h (remifentanil: 0.5 µg/kg/min in the remifentanil group, fentanyl: 2 µg/kg/h in the fentanyl group) in the titration and maintenance phases. The infusion rate of the study drug was gradually reduced toward weaning from the ventilator while observing the general condition of the patient. The infusion rate was reduced by up to 25% until the end of treatment, at intervals of at least 10 min, and weaning from the ventilator was carried out after the end of administration. If the infusion rate was ≤ 1 mL/h (remifentanil: ≤ 0.025 µg/kg/min in the remifentanil group, fentanyl: ≤ 0.1 µg/kg/h in the fentanyl group) at the start of infusion reduction, or if the infusion rate of ≤ 1 mL/h (remifentanil: ≤ 0.025 µg/kg/min in the remifentanil group, fentanyl: ≤ 0.1 µg/kg/h in the fentanyl group) was reached during the infusion reduction, the administration was terminated without further reduction. However, further infusion reduction was allowed depending on the condition of the patients. The study drug was gradually switched to other analgesics for pain management as the infusion rate decreased. A 24-h follow-up period was provided after the end of administration. A sedative was allowed to be used as needed. The target sedation level was RASS ≤ 0, with an intended level of -2 to 0, since analgesia could not be assessed at a RASS of ≤ -3. If both the analgesia and sedation levels did not meet each target level, the analgesia level was preferred. Temporary use of muscle relaxants was permitted as needed, but continuous use was prohibited. Systemic analgesics were prohibited during the treatment and observation periods. Use of nalmefene was prohibited for one week before titration, and during treatment. Epidural or intrathecal administration of local anesthetics or analgesics, and nerve block were prohibited from the start of dosing to the start of dose reduction with the intention of weaning from the ventilator.

### Efficacy and safety assessments

Analgesia was assessed using BPS. However, if the BPS-based assessment was inappropriate because the patients was clearly conscious, the NRS was used. Analgesia was assessed at pre-dosing and at 5, 10, 15, 20, and 30 min, 1, 2, and every 2 h after the start of dosing, 5-min prior to termination of dosing, and 10, 20, 30 min and 1 h after the termination of dosing. When the infusion rate was changed, rescue fentanyl was administered or the sedative infusion rate was changed, analgesia was assessed within 5 min before the change or the start of dosing, and 5, and 15 min after the change or the start of dosing. Sedation was assessed with the RASS using the same schedule of analgesia assessment. The validity/accuracy of the RASS/BPS/NRS assessment was ensured by requiring all investigators, subinvestigators and nurses to take training prior to conducting any assessment related to this trial.

The primary endpoint was the proportion of the patients who did not require rescue administration of analgesia between the start of dosing and the start of dose-reduction toward weaning from the ventilator. The secondary endpoints were the number of rescue dose of fentanyl and the total dose of such doses, doses of sedatives used during treatment, proportion of time where BPS was maintained ≤ 5 or NRS was ≤ 3, proportion of time where RASS was maintained at ≤ 0, the proportion of time where RASS was between -2 and 0, duration from the end of dosing to weaning from the ventilator, duration from the start of dose-reduction to weaning from the ventilator, the infusion rate of the study drug (mean, minimal, and maximum) and duration of treatment.

Safety was assessed according to adverse events (AEs), laboratory tests including hematology, blood biochemistry, and urinalysis, vital signs, percutaneous oxygen saturation (SpO_2_), end-tidal carbon dioxide (ETCO_2_), blood gas analyses including partial pressure of arterial oxygen, partial pressure of arterial carbone dioxide, arterial oxygen saturation, HCO_3_^−^, and pH, body weight, and 12-lead electrocardiogram (ECG). Vital signs (blood pressure, heart rate and respiratory rate), SpO_2_ and ETCO_2_ were assessed at the same time points as the BPS/NRS up to 1 h after the termination of study drug dosing, and at 6 and 24 h after the end of dosing.

### Pharmacokinetics

PK was assessed in 24 patients whose body mass index was < 25 in the remifentanil group. Arterial blood samples for pharmacokinetic analysis were collected 1 h after the start of dosing, immediately before the start of dose reduction leading into weaning from the ventilator, at the end of dosing, and 1, 2, 3, 5, 7, 10, 20, and 60 min after the end of dosing. Remifentanil concentrations in arterial blood were measured using a liquid chromatography–tandem mass spectrometry method [19]. The quantification limit was < 0.05 ng/mL.

### Statistical analysis

The sample size was established based on the expected proportion of patients who would not require rescue fentanyl. We conservatively expected 85% of patients would not need a rescue analgesic in the remifentanil and fentanyl groups based on the reported efficacy proportion of ≥ 95% [8]. Placebo effect was assumed to be 50% based on the proportions of non-rescue analgesics in the clinical studies of sedatives [20–23]. The non-inferiority margin for this study was set at 15%. To show non-inferiority of remifentanil to fentanyl under these conditions, with a one-sided alpha level of 2.5% and 80% of power, each treatment group should consist of 90 participants. In addition, the number of participants needed to verify that the effective proportion of remifentanil exceeded a threshold of 70% with the expected efficacy of 85%, significance level 5% on both sides, and power of 80% was 65. Therefore, this study planned to enroll 90 patients/group (total of 180 patients). For PK assessment, 20 participants were selected in order to calculate the PK parameters.

Safety analysis was performed for subjects in the safety analysis set (SS), and efficacy was analyzed primarily for the full analysis set (FAS) and secondarily in the per protocol set (PPS). The SS consisted of subjects who received the study drug at least once excluding those who were good clinical practice (GCP) non-compliant. The FAS included the SS subjects excluding those who had no primary data-assessment endpoint. The PPS was defined as the subset of subjects in the FAS excluding those with deviations from the protocol determined to affect efficacy assessment.

For the primary endpoint, the proportion of patients who did not require rescue administration of analgesia and its 95% confidence interval (CI) were calculated in each group. In the remifentanil group, the lower limit of the 95% CI was compared with the threshold of 70%. The difference and its 95% CI (Wald method) in the effective proportions between the groups was calculated. Non-inferiority of remifentanil to fentanyl was verified by the Δ-addition method (non-inferiority margin Δ = 15%). For sensitivity analysis, the primary endpoint was evaluated in the PPS population. The primary endpoint was also evaluated in the subpopulation that included sex (male and female) and age (< 65 years and ≥ 65 years), type of ICU patients (internal medicine patients and post-operative ones), and concomitant sedation (dexmedetomidine and propofol). For secondary endpoints, descriptive statistics were shown for each group and the 95% CI of the mean was calculated. In addition, the difference in the mean and its 95% CI were calculated, and compared between the groups using t-test. The number of doses in the remifentanil group was compared with that in the fentanyl group by the Wilcoxon rank sum test.

For safety assessment, events that occurred after administration of the study drug were analyzed. The number of AEs or adverse drug reactions (ADRs), defined as AEs other than those for which a causal relationship was not ruled out, and number of subjects who experienced AEs were shown by group. The difference in AEs and ADRs between the groups were analyzed by Fisher’s exact test. AEs were coded using the Medical Dictionary for Regulatory Activities Japanese edition ver. 23.1.

PK assessment, descriptive statistics including number of subjects, mean, standard deviation (SD) for remifentanil concentration in arterial blood were shown for each measurement time. Descriptive statistics including number of subjects, mean, median, geometric mean, SD, coefficient of variation (CV), minimum, and maximum of the PK parameters [non-compartment model: elimination half-life (t_1/2_), area under the concentration–time curve from time zero to time t (AUC_0-t_), AUC from time zero to infinity (AUC_0-inf_), maximum concentration (C_max_), total body clearance (CL), distribution volume at steady state (V_ss_)] were calculated.

For missing data, no data complementation was performed related to efficacy, safety and pharmacokinetic analysis. All statistical tests were performed using a two-sided significance level of 0.05. All analyses were performed using SAS version 9.4 (SAS Institute Inc., Cary, NC, USA).

## Results

### Patient disposition and baseline characteristics

**Fig. 1:**
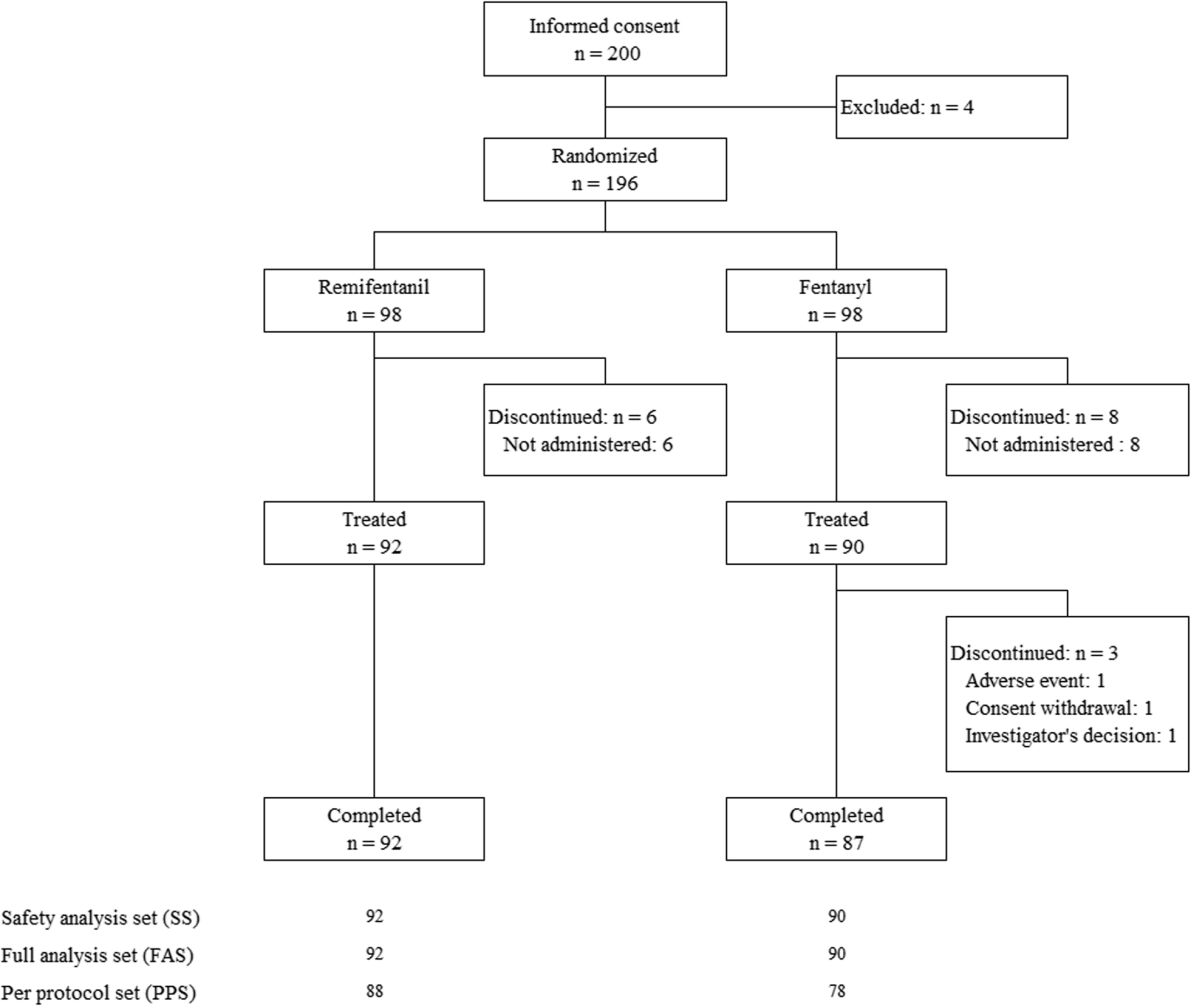
Enrollment, randomization, and treatment assignment

Among 200 patients who gave informed consent, 196 patients who met the eligibility criteria were randomized to receive either remifentanil (n = 98) or fentanyl (n = 98), and 182 patients received one or more doses of the study drug. A total of 179 patients completed the study (Fig. 1). The SS and FAS population was 182 after excluding 14 patients who did not receive the study drug. Baseline patient characteristics in the FAS population were similar across treatment groups (Table 1). The mean minimum infusion rate of remifentanil was 0.024 μg/kg/min (Table 2). Among five of the 92 patients, a infusion rate lower than the specified initiation rate of 0.025 μg/kg/min of remifentanil was administered; infusion reduction after the end of administration (two), infusion reduction due to sufficient effect (two), and AE (one).

**Table 1:** Patient characteristics (FAS)

		Remifentanil N = 92	Fentanyl N = 90	Total N = 182
Age (y)	Mean ± SD	68.5 ± 10.9	65.9 ± 13.2	67.2 ± 12.1
Gender	Male, n (%)	69 (75.0%)	72 (80.0%)	141 (77.5%)
BMI (kg/m^2^)	Mean ± SD	21.54 ± 3.69	22.41 ± 3.86	21.97 ± 3.79
ICU	Internal medicine, n (%)	10 (10.9%)	15 (16.7%)	25 (13.7%)
	Post-operation, n (%)	82 (89.1%)	75 (83.3%)	157 (86.3%)
ASA (post-operation patients) n (%)	I	7 (8.5%)	7 (9.3%)	14 (8.9%)
	II	62 (75.6%)	48 (64.0%)	110 (70.1%)
	III	13 (15.9%)	20 (26.7%)	33 (21.0%)
Duration of anesthesia (h) (post-operation patients) *	Mean ± SD	10.95 ± 3.64 (82)	10.73 ± 3.19 (75)	10.85 ± 3.42 (157)
Operation time (h) (post-operation patients) *	Mean ± SD	9.52 ± 3.43 (82)	9.26 ± 3.12 (75)	9.40 ± 3.28 (157)
Analgesia assessment				
Method	BPS, n (%)	88 (95.7%)	89 (98.9%)	177 (97.3%)
	NRS, n (%)	4 (4.3%)	1 (1.1%)	5 (2.7%)
BPS *	Mean ± SD	3.6 ± 1.1 (88)	3.5 ± 1.1 (89)	3.6 ± 1.1 (177)
NRS *	Mean ± SD	2.3 ± 3.2 (4)	0.0 (1)	1.8 ± 2.9 (5)
Sedation assessment				
RASS, n (%)	0	31 (33.7%)	24 (26.7%)	55 (30.2%)
	− 1	30 (32.6%)	27 (30.0%)	57 (31.3%)
	− 2	31 (33.7%)	38 (42.2%)	69 (37.9%)
	− 3	0	0	0
	− 4	0	0	0
	− 5	0	1 (1.1%)	1 (0.5%)

*ASA* American Society of Anesthesiologist Physical Status, *BMI* body mass index, *BPS* behavioral pain scale, *FAS* full analysis set, *ICU* intensive care unit, *NRS* numerical rating scale, *RASS* Richmond agitation-sedation scale, *SD* standard deviation,

^*^Data are shown as Mean ± SD (n)

**Table 2:** Infusion rate and administration period of investigational drug

	Remifentanil N = 92	Fentanyl N = 90
Infusion rate-mean Remifentanil: µg/kg/min Fentanyl: µg/kg/h	0.046 ± 0.036 (0.02, 0.24)	0.215 ± 0.191 (0.10, 1.17)
Infusion rate-minimum Remifentanil: µg/kg/min Fentanyl: µg/kg/h	0.024 ± 0.002 (0.01, 0.025)	0.100 ± 0.001 (0.09, 0.10)
Infusion rate-maximum Remifentanil: µg/kg/min Fentanyl: µg/kg/h	0.056 ± 0.054 (0.025, 0.34)	0.321 ± 0.644 (0.10, 6.00)
Dosing duration (h)	12.37 ± 14.58 (6.0, 119.5)	12.59 ± 16.26 (3.6, 121.9)

Data are shown as Mean ± SD (Min, Max)

### Efficacy

The mean ± SD of the treatment period (h) in the remifentanil and fentanyl groups was 12.37 ± 14.58 and 12.59 ± 16.26, respectively (Table 2). For the primary endpoint, the proportion of the patients who did not require a rescue dose of fentanyl was 100% (92/92) (95% CI, 96.1–100) in the remifentanil group and 97.8% (88/90) (95% CI, 92.2–99.7) (Table 3). The lower limit of the 95% CIs in the both groups (96.1% for the remifentanil group and 92.2% for the fentanyl group) exceeded the threshold of 70%. The difference in the proportions and the 95% CIs between the groups was 2.2% and − 0.8–5.3. The null hypothesis that the proportion in the remifentanil group was more than 15% inferior to that of the fentanyl group was rejected, and non-inferiority of remifentanil to fentanyl was verified (*p* < 0.0001). In the PPS population, no patients in either groups required the use of rescue analgesics. Non-inferiority of remifentanil to fentanyl was observed in the subpopulation analysis (Additional file 1: Table S2). With respect to the secondary endpoints, remifentanil showed a similar effect to fentanyl in terms of the number of doses and total dose of rescue analgesics, doses of sedative during the treatment, the proportion of time with BPS ≤ 5 or NRS ≤ 3, the proportion of time with RASS ≤ 0, the proportion of time with a RASS maintained between − 2 and 0, duration from the end of dosing to weaning from the ventilator, duration from the start of dose-reduction to weaning from the ventilator, infusion rate, and dosing duration (Table 3). Although there was no significant difference, the dose of sedatives in the remifentanil group tended to be slightly lower than that in the fentanyl group. The maximum infusion rate in the remifentanil group was in the range of 0.025 to 0.34 μg/kg/min (Table 2).

**Table 3:** Summary of efficacy (FAS)

	Remifentanil N = 92	Fentanyl N = 90	Difference [95% confidence interval]	*p*-Value
*Primary endpoint*				
Proportion of the patients who did not require rescue analgesics (%)*	100.0 [96.1, 100.0]	97.8 [92.2, 99.7]	2.2 [ − 0.8, 5.3]	< 0.0001
No. of patients who did not require rescue analgesics	92	88	–	–
*Secondary endpoints*				
No. of rescue analgesics doses^†^	0.00 ± 0.00 [–, –]	0.03 ± 0.23 [− 0.02, 0.08]	–	0.1516
Total dose of rescue analgesics^†^ (µg/kg)	0.00 ± 0.00 [–, –]	0.02 ± 0.12 [− 0.01, 0.04]	− 0.02 [− 0.04, 0.01]	0.1853
Concomitant use of sedatives				
Yes, n (%)	78 (84.8%)	78 (86.7%)		
Propofol (mg)^†^	435.66 ± 744.62 [281.45, 589.86]	693.22 ± 1613.79 [355.22, 1031.22]	− 257.56 [-623.89, 108.77]	0.1670
Dexmedetomidine (µg)^†^	158.51 ± 186.92 [119.80, 197.22]	236.47 ± 494.56 [132.89, 340.06]	− 77.96 [− 186.87, 30.95]	0.1595
Proportion of duration maintained BPS ≤ 5 or NRS ≤ 3 (%)^†^	99.16 ± 2.60 [98.62, 99.70]	98.50 ± 3.44 [97.78, 99.22]	0.66 [− 0.24, 1.55]	0.1486
Proportion of duration maintained RASS ≤ 0 (%)^†^	99.54 ± 1.53 [99.22, 99.85]	99.07 ± 3.69 [98.30, 99.84]	0.47 [− 0.36, 1.29]	0.2640
Proportion of duration maintained RASS = − 2 to 0 (%)^†^	89.66 ± 24.24 [84.64, 94.68]	83.86 ± 28.92 [77.81, 89.92]	5.80 [− 2.00, 13.60]	0.1439
Duration from the end of dosing to weaning from the ventilator (h)^†^	1.68 ± 4.31 [0.79, 2.57]	1.17 ± 2.68 [0.61,1.74] ‡	0.51 [− 0.55, 1.57]	0.3481
Duration from the start of dose-reduction toward the ventilator weaning (h)^†^	1.89 ± 4.29 [1.00, 2.78]	1.45 ± 2.78 [0.86, 2.04] ‡	0.44 [− 0.63, 1.51]	0.4157

The difference between the mean values was analyzed by the t-test. Number of doses between the groups was compared by the Wilcoxon rank sum test

*BPS* behavioral pain scale, *FAS* full analysis set, *Max* maximum, *Med* median, *Min* minimum, *NRS* numerical rating scale, *RASS* Richmond agitation-sedation scale, *SD* standard deviation

^*^Data are shown as percentages [95% confidence interval]

^†^Data are shown as Mean ± SD [95% confidence interval]

^‡^n = 88

### Safety

**Fig. 2:**
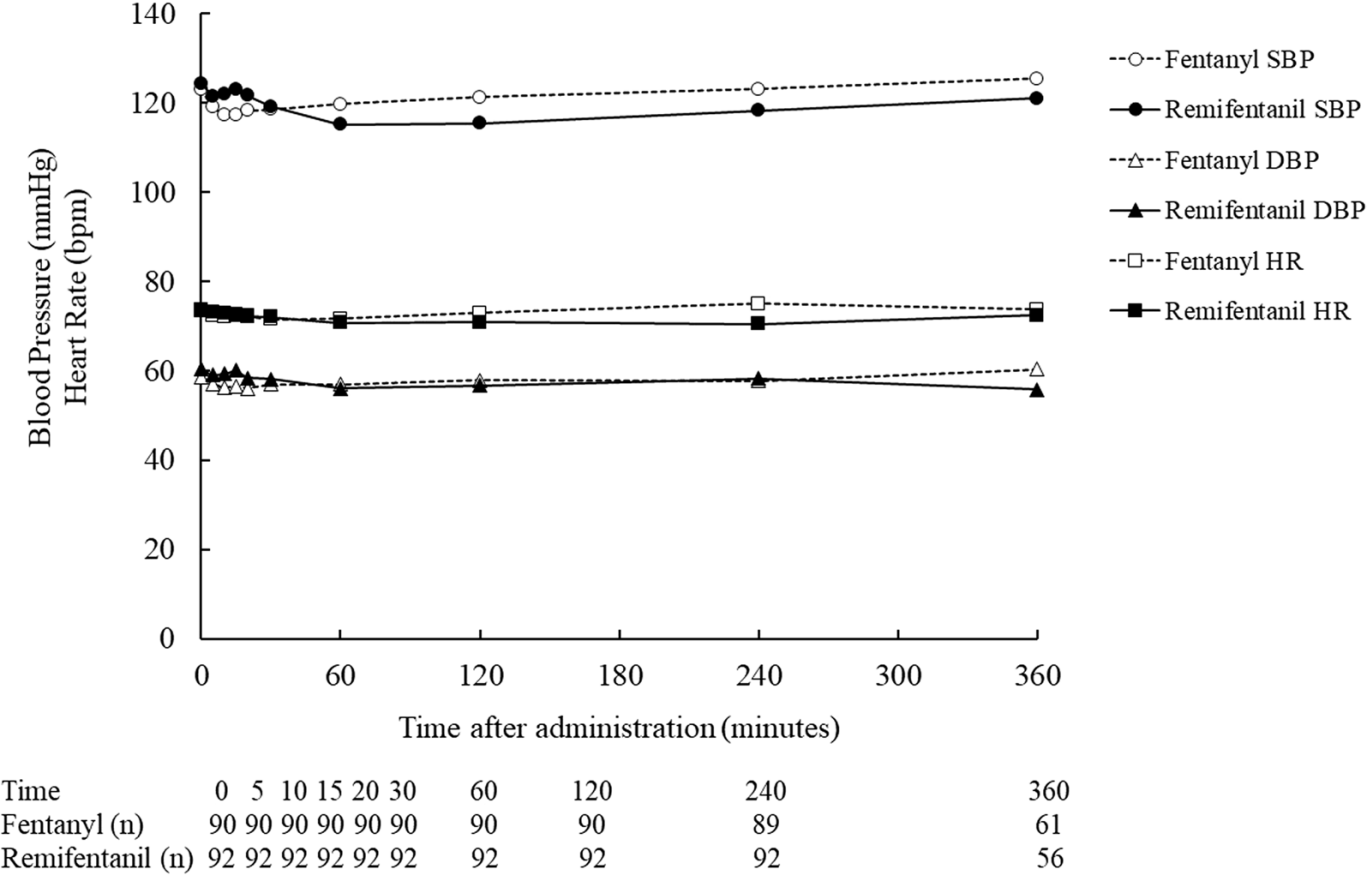
Mean blood pressure and heart rate over time. Blood pressure and heart rate were monitored during the administration of the study drug and measured at pre-dosing and at 5, 10, 15, 20, and 30 min, 1, 2, and every 2 h after the start of dosing. Vital signs were also measured before and after a change in infusion rate of study drug, rescue fentanyl administration, a change in sedative infusion rate and a termination of dosing of study drug, but these values are not included in the figure. *DBP* diastolic blood pressure, *HR* heart rate, *SBP*  systolic blood pressure

The incidence of any AEs in the remifentanil and fentanyl groups was 34 of 92 patients (37.0%) and 34 of 90 patients (37.8%), respectively (Table 4). ADRs occurred 12 of 92 patients (13.0%) in the remifentanil group and 15 in 90 patients (16.7%) in the fentanyl group. There was no difference in the incidence of AEs and ADRs between the remifentanil and fentanyl groups. Severity of most AEs was mild to moderate, except one patient in the remifentanil group who experienced a severe graft complication, and two patients in the fentanyl group who experienced severe atrial fibrillation or thrombosis. There was no difference in the severity of AEs and ADRs between the groups. No death was reported. Two serious AEs (SAEs) excluding death were reported in each group; graft complications and vocal cord paralysis in the remifentanil group, and fibrillation and thrombosis in the fentanyl group. None of the SAEs was considered related to the study drug, and the outcomes were recovery or amelioration. There was no AEs that led to discontinuation of the study drug in any of the groups. There were no obvious changes in heart rate or blood pressure from before administration, nor were there any differences between the treatment groups (Fig. 2). Laboratory tests, SpO_2_, ETCO_2_, and blood gas did not change significantly in the 24 h after the end of dosing in either group. No significant changes were observed after any change in the infusion rate of the study drug.

**Table 4:** Summary of safety (SS)

	Remifentanil N = 92	Fentanyl N = 90
	n (%)	n (%)
AEs	34 (37.0)	34 (37.8)
Serious AEs except for death	2 (2.2)	2 (2.2)
AEs leading to discontinuation	0 (0.0)	0 (0.0)
Death	0 (0.0)	0 (0.0)
Adverse drug reactions	12 (13.0)	15 (16.7)
Serious adverse drug reactions	0 (0.0)	0 (0.0)
Serious adverse drug reactions leading to discontinuation	0 (0.0)	0 (0.0)
AEs (≥ 2% in any group)		
Anaemia	0 (0.0)	2 (2.2)
Hypokalaemia	3 (3.3)	1 (1.1)
Delirium	3 (3.3)	4 (4.4)
Insomnia	2 (2.2)	3 (3.3)
Restlessness	1 (1.1)	3 (3.3)
Atrial fibrillation	3 (3.3)	1 (1.1)
Bradycardia	2 (2.2)	1 (1.1)
Hypertension	3 (3.3)	0 (0.0)
Hypotension	7 (7.6)	7 (7.8)
Bradypnoea	2 (2.2)	0 (0.0)
Nausea	3 (3.3)	6 (6.7)
Hepatic function abnormal	2 (2.2)	0 (0.0)
Rash	2 (2.2)	0 (0.0)
Pain	2 (2.2)	1 (1.1)
Post procedural hypotension	0 (0.0)	2 (2.2)
Adverse drug reactions (≥ 2% in any group)		
Hypotension	3 (3.3)	3 (3.3)
Bradypnoea	2 (2.2)	0 (0.0)
Nausea	1 (1.1)	3 (3.3)
Post procedural hypotension	0 (0.0)	2 (2.2)

MedDRA/J Version 23.1

*AE* adverse event, *SS* safety analysis set

The clinically significant changes in the ECG were atrial fibrillation in one patient in the remifentanil group, and atrial fibrillation, ventricular arrhythmia, and supraventricular extrasystole in one patient in the fentanyl group. Atrial fibrillation in the remifentanil group was a non-SAE with mild severity and no causal relationship to the study drug and any recovery outcome.

### Pharmacokinetics

**Fig. 3:**
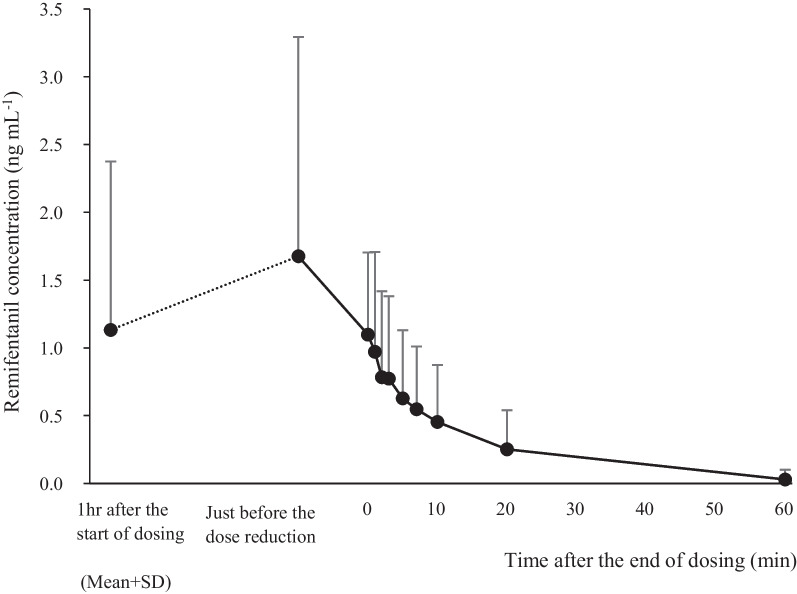
Remifentanil concentrations in arterial blood. Remifentanil concentrations in arterial blood were measured 1 h after the start of dosing, immediately before the start of dose reduction leading into weaning from the ventilator, at the end of dosing, and 1, 2, 3, 5, 7, 10, 20 and 60 min after the end of dosing. Concentrations are indicated by mean + standard deviation

The mean ± SD of the treatment period in the PK populations was 17.36 ± 23.94 h. The arterial blood concentration of remifentanil, active ingredient of remifentanil was measured in 24 patients in the remifentanil group (Fig. 3). The remifentanil concentration (mean ± SD) decreased from 1.676 ± 1.618 ng / mL immediately before the start of the dose reduction toward weaning from the ventilator to 1.098 ± 0.6059 ng / mL at the end of administration. The concentration 60 min after the end of administration was 0.02875 ± 0.07245 ng/mL and among 20 of 24 patients, the concentration was less than the 0.05 ng/mL lower limit of quantification. The PK parameters (mean ± SD) calculated based on the non-compartment model are shown in Table 5.

**Table 5:** Pharmacokinetic parameters of remifentanil

	t_1/2_	AUC_0-t_	AUC_0-inf_	C_max_	CL	V_ss_
	(min)	(min*ng/mL)	(min*ng/mL)	(ng/mL)	(mL/min/kg)	(mL/kg)
No. of patients	24	24	24	24	24	22
Mean ± SD	16.97 ± 19.81	1718 ± 2938	1726 ± 2937	1.803 ± 1.594	41.65 ± 19.70	9600 ± 16,870
(Min, Med, Max)	(4.20, 9.895, 93.7)	(85.6, 400.5, 11,800)	(87.0, 409.0, 11,800)	(0.539, 1.150, 6.08)	(19.3, 34.35, 106.0)	(292, 2125, 72,500)
Geometric mean	11.95	664.9	680.8	1.384	38.26	2960
Coefficient of variation	1.167	1.71	1.702	0.884	0.473	1.758

*AUC*_*0-t*_ area under the concentration–time curve from time zero to time t, *AUC*_*0-inf*_ AUC from time zero to infinity, *CL* total body clearance, *C*_*max*_ maximum concentration, *t*_*1/2*_ elimination half-life, *V*_*ss*_ distribution volume at steady state

## Discussion

In Japan, remifentanil had been approved for analgesia during induction and maintenance of general anesthesia and maintenance of general anesthesia for pediatric patients and adults. On the other hand, in the United States and European countries, continuing analgesia until entering the ICU or PACU after general anesthesia, and analgesia in the ICU have been approved. In this multicenter, randomized, double-blinded, parallel-group, fentanyl-controlled non-inferiority phase 3 study, we demonstrated that continuous intravenous administration of remifentanil was as effective as fentanyl for analgesia in Japanese patients requiring respiratory management in intensive care, in which the primary endpoint was the proportion of patients who did not require rescue analgesics. In this study, patients were managed with analgesia-first sedation while in the ICU, as is usual in clinical practice, and it was expected that pain would be well controlled even before the start of study drug administration. Therefore, the primary endpoint of the study was set the proportion of patients who did not require rescue analgesics, not the proportion of the patients in the target analgesic range. Furthermore, consistent non-inferiority of remifentanil to fentanyl was observed in the secondary endpoints including number of doses and total dose of the rescue analgesics, dose of sedative during the treatment, the proportion of time with maintenance of BPS ≤ 5 or NRS ≤ 3, the proportion of time with maintenance of RASS ≤ 0, the proportion of time with maintenance of RASS − 2 to 0, duration from the end of dosing to weaning from the ventilator, and duration from the start of dose-reduction to weaning from the ventilator, confirming a robust effect of remifentanil in patients in the ICU setting.

Several studies have suggested that analgesia-first sedation with remifentanil reduce requirements of sedative drug and contribute to improved clinical outcomes, such as decrease duration of ventilation and ICU length of stay [8, 9, 12, 24, 25]. In the present study, similar to these reports, there was a trend towards less sedative drug use in the remifentanil group compared with the fentanyl group (not significant), while there was no difference in duration from the end of dosing to weaning from the ventilator and duration from the start of dose-reduction to weaning from the ventilator between the two groups. Our study required frequent observations of vital signs, SpO_2_, ETCO_2_, analgesia and sedation assessment, and frequent blood sampling for pharmacokinetic analysis before and after completion of study drug administration. Therefore, even if the patient was stable enough to be weaned from ventilation, observation and assessment for the study was considered a priority from the end of administration to weaning, and weaning from ventilator was performed only after all necessary observations for the study had been completed. This may have resulted in results of our study that did not reflect real-world clinical practice with regard to outcomes related to weaning from ventilator.

The dose of remifentanil used in the current indications for adults are 0.5 μg/kg/min continuous intravenous infusion for induction, and 0.25 μg/kg/min for maintenance of general anesthesia. The maximum infusion dose is 2.0 μg/kg/min. In European countries, the initial infusion dose is 0.1–0.15 μg/kg/min and increased by 0.025 μg/kg/min at an interval ≥ 5 min in the range of 0.006–0.74 μg/kg/min in mechanically ventilated patients in intensive care [26]. Bolus doses of remifentanil are not recommended in the intensive care setting [26, 27]. In this study, administration of remifentanil was started at an infusion rate of 0.025 µg/kg/min in all 92 patients in the remifentanil group, which was one quarter of that used in European countries [26], suggesting 0.025 µg/kg/min is acceptable for Japanese patients in the ICU, from a safety point of view. The maximum infusion rate of remifentanil was in the range of 0.025–0.34 µg/kg/min to achieve the targeted analgesia and sedation levels, which was less than the 0.5 µg/kg/min specified in our study as a maximum infusion rate and much lower than the maximum infusion rate of 0.74 µg/kg/min in European countries [26]. In this study, although no rescue doses of fentanyl were given in remifentanil groups, the mean rate of remifentanil administration (0.046 ± 0.036 μg/kg/min) was not higher than in other reports [8, 9, 12]. This may be due to the fact that in this study, remifentanil was used for pain management and once the target analgesic level was reached, the sedation level was managed with sedatives, whereas in other reports, remifentanil was used not only for analgesia but also for sedation management.

The infusion rate of the study drug was gradually reduced as weaning from the ventilator was approached, and was terminated prior to weaning from the ventilator due to the risk of serious respiratory depression [28]. Duration of treatment in this study was designed to be 6 h–10 days. The six hours was set to evaluate the effectiveness of remifentanil sufficiently. In the United Kingdom, use of remifentanil for mechanically ventilated patients in the ICU is not recommended for a treatment duration greater than three days [26], as remifentanil has only been studied for up to three days [8, 9, 29, 30]. However, Karabinis et al. [11] reported mechanically ventilated patients with acute brain injury or who had undergone neurosurgery were evaluated for up to 5 days. In two longer-term studies, patients were mechanically ventilated for up to 10 days without any safety concerns [12]. Therefore, the upper limit was set to 10 days. The actual mean treatment duration was 12.37 ± 14.58 h and the maximum was 119.5 h (5.0 days), which were similar to those where fentanyl was used. Taken together, remifentanil can be used safely and effectively by starting at an infusion rate of 0.025 μg/kg/min and adjusting the infusion rate within a range up to 0.5 μg/kg/min, for up to at least 5 days.

Regarding the incidences of any AEs and ADRs, no notable differences were observed between the remifentanil and fentanyl groups. None of the SAEs was considered to be related to the study drug. There was no AEs leading to discontinuation of the study drug in any of the groups. None of the patients in the remifentanil group had AEs within 30 min of starting the infusion. It has been reported [4] that remifentanil was generally well tolerated in mechanically ventilated patients in the ICU as observed in this study. The most commonly occurring AEs were related to its μ-opioid agonist properties such as hypotension and bradycardia.

The efficacy and safety of remifentanil when administered for more than 5 days could not be evaluated in this study. Withdrawal syndrome is one of the most concerning symptoms of long-term opioid use. Delvaux et al. reported the cases of severe withdrawal syndrome after remifentanil administration of between 2 and 33 days’ duration [31]. However, these events occurred with sudden reductions and discontinuations of remifentanil dose, whereas our study stipulated that the dose be gradually tapered off before the end of treatment. The risk of withdrawal symptoms is considered to be low if the administration is tapered as in this study without sudden discontinuation, and the current package insert of remifentanil recommend a gradually tapering the infusion rate until the end of treatment in order to prevent the opioid withdrawal syndrome [1].

Tolerance is another concern with long-term opioid administration. A study in which remifentanil was administered for up to 10 days at an average dosing rate of 0.32 μg/kg/min reported no clinical evidence of suspected development of tolerance to remifentanil, such as escalated remifentanil requirements or post-infusion opioid requirements [12]. Furthermore, reports of up to 28 days of remifentanil administration did not show an increase in the mean rate of administration of the remifentanil over time suggestive of tolerance [32]. Review article by Yu et al. [33] found that of the 12 studies examining remifentanil tolerance, only one study clearly suggested tolerance and 4 studies inferred its presence. While the remaining seven studies reported negative or inconclusive results, thus leaving no consensus on the development of tolerance to remifentanil. In order to fully evaluate the development of tolerance to remifentanil in long-term use, future studies or reports are awaited.

Several PK studies of remifentanil in adults are reported [34–42], but as far as we know, there is only one report in the ICU patients [10].We note that this is the first PK study of remifentanil in Japanese patients mechanically ventilated in ICU. This study demonstrated within 10 min that blood remifentanil concentration decreased to almost 50% of the initial concentration at the end of administration, although individual variability was large (Fig. 3). The t_1/2_ estimated by the non-compartment model in our study was 16.97 ± 19.81 min, and CL was 41.65 ± 19.7 mL/min/kg, which was not notably different from a previous report in ICU patients with normal/mild impaired renal function (11.4 ± 7.24 min and 44.3 ± 14.4 mL/min/kg, respectively) [10]. In addition, the t_1/2_ β phase (t_1/2β_) and CL reported in Japanese patients under general anesthesia ranges from 12.62 to 16.48 min and 44.8 to 55.4 mL/min/kg, respectively [42]. Although there are differences in the method of estimating PK parameters, the PK profile, particularly, t_1/2_ and CL in ICU patients was similar to those seen in patients under general anesthesia despite the treatment period in ICU patients being longer than the duration of general anesthesia.

There are, however, some limitations to this study. First, although the use of analgesia-based sedation with remifentanil were compared with a comparator, no standard primary endpoint was available in the clinical studies of remifentanil in mechanically ventilated patients in the ICU. An alternate reported primary endpoint was duration of mechanical ventilation. In this study, the proportion of patients who did not require analgesic rescue was evaluated as the primary endpoint. However, it was noteworthy that the efficacy of remifentanil in mechanically ventilated patients in the ICU was at least similar to fentanyl and morphine with a variety of types of assessment. Second, the maximum duration of treatment was set to 10 days, but the observed duration was 5 days. The efficacy and safety of remifentanil over 5 days should be evaluated if longer-duration is used in the real world.

## Conclusion

This study showed that continuous intravenous administration of remifentanil was as effective as fentanyl for analgesia in mechanically ventilated Japanese patients in intensive care. In terms of safety, no characteristics different from those of fentanyl were observed. Rapid offset of action following discontinuation was confirmed in Japanese patients. remifentanil is an opioid that is easy to control and can be used safely for pain management in mechanically ventilated patients in the ICU.

### Supplementary Information

**Additional file 1: Table S1.** List of hospitals participating in the study. **Table S2.** Subgroup analysis of the primary endpoint.

## Data Availability

The study protocol and statistical analysis plan (Japanese edition only) will be shared with those who request data sharing. Requests for data should be directed to the corresponding author. Requests will be reviewed, and scientifically sound proposals will be approved by the sponsor (Maruishi Pharmaceutical Co., Ltd.). In addition, an agreement for data sharing needs to be contracted between data requestors and the sponsor. Data will be shared up to two years after article publication.

## Abbreviations

ADR: Adverse drug reaction
AE: Adverse event
ASA: American Society of Anesthesiologist Physical Status
AUC_0-t_: Area under the concentration–time curve from time zero to time t
AUC_0-inf_: AUC from time zero to infinity
BMI: Body mass index
BPS: Behavioral pain scale
CI: Confidence interval
CL: Total body clearance
C_max_: Maximum concentration
CV: Coefficient of variation
DBP: Diastolic blood pressure
ECG: Electrocardiogram
ETCO_2_: End tidal carbone dioxide
FAS: Full analysis set
GCP: Good Clinical Practice
HR: Heart rate
ICU: Intensive care unit
NRS: Numerical rating scale
PACU: Postoperative care unit
PK: Pharmacokinetics
PPS: Per protocol set
RASS: Richmond Agitation-Sedation Scale
SAE: Serious AEs
SBP: Systolic blood pressure
SD: Standard deviation
SpO_2_: Percutaneous oxygen saturation
SS: Safety analysis set
t_1/2_: Elimination half-life
t_1/2β_: t_1/2_ β phase
V_ss_: Distribution volume at steady state
