# Supplementary material for: Efficacy, safety, and pharmacokinetics of MR13A11A, a generic of remifentanil, for pain management of Japanese patients in the intensive care unit: a double-blinded, fentanyl-controlled, randomized, non-inferiority phase 3 study
Source: J Intensive Care. 2023 Nov 13;11:51. doi: 10.1186/s40560-023-00698-9 (PMC10641973; doi:10.1186/s40560-023-00698-9)
Supplement: Supplementary file 1 — Additional file 1: Table S1. List of hospitals participating in the study. Table S2. Subgroup analysis of the primary endpoint. [file 40560_2023_698_MOESM1_ESM.docx]

**Additional Information**

**Efficacy, safety, and pharmacokinetics of MR13A11A, a generic of remifentanil, for pain management of Japanese patients in the intensive care unit; a double-blinded, fentanyl-controlled, randomized, non-inferiority phase 3 study**

**Table S1** List of hospitals participating in the study

**Table S2** Subgroup analysis of the primary endpoint

**Table S1** List of hospitals participating in the study

|  |  |
| --- | --- |
|  |  |
| Tokyo Bay Urayasu ichikawa Medical Center | National Hospital Organization Tokyo Medical Center |
| Tokyo Medical and Dental University Hospital | National Hospital Organization Yokohama Medical Center |
| Shonan Kamakura General Hospital | Hamamatsu University School of Medicine |
| University Hospital, Kyoto Prefectural University of Medicine | Kansai Medical University Hospital |
| Osaka University Hospital | Sakai City Medical Center |
| Osaka General Medical Center | Wakayama Medical University Hospital |
| Kobe City Medical Center General Hospital | Kokura Memorial Hospital |
| Shimane University Hospital | Tokyo Medical University Hachioji Medical Center |
| Fujita Health University Hospital | Medical Research Institute Kitano Hospital |
| Saga University Hospital | Tokyo Medical University Hospital |
| Kanazawa University Hospital | Fukui University Hospital |
| Shiga University of Medical Science Hospital | Tottori University Hospital |
| Nara Prefecture General Medical Center | Hyogo Medical University Hospital |
| Yokohama City Minato Red Cross Hospital | Kobe University Hospital |
| Nara Medical University Hospital |  |
|  |  |

**Table S2** Subgroup analysis of the primary endpoint

|  |  | Group | N | No-rescue use | |  | Difference between the groups | |  |
| --- | --- | --- | --- | --- | --- | --- | --- | --- | --- |
|  |  |  |  | n (%) | 95% CI |  | Difference | 95% CI | *p*-value |
| Age | <65 | Remifentanil | 32 | 32 (100.0) | [89.1, 100.0] |  | 2.9 | [-2.7, 8.4] | <0.0001 |
|  |  | Fentanyl | 35 | 34 (97.1) | [85.1, 99.9] |  |  |  |  |
|  | ≥65 | Remifentanil | 60 | 60 (100.0) | [94.0, 100.0] |  | 1.8 | [-1.7, 5.3] | <0.0001 |
|  |  | Fentanyl | 55 | 54 (98.2) | [90.3, 100.0] |  |  |  |  |
| Sex | Male | Remifentanil | 69 | 69 (100.0) | [94.8, 100.0] |  | 1.4 | [-1.3, 4.1] | <0.0001 |
|  |  | Fentanyl | 72 | 71 (98.6) | [92.5, 100.0] |  |  |  |  |
|  | Female | Remifentanil | 23 | 23 (100.0) | [85.2, 100.0] |  | 5.6 | [-5.0, 16.1] | <0.0001 |
|  |  | Fentanyl | 18 | 17 (94.4) | [72.7 ,99.9] |  |  |  |  |
| ICU | Internal medicine | Remifentanil | 10 | 10 (100.0) | [69.2, 100.0] |  | 6.7 | [-6.0, 19.3] | 0.0004 |
|  |  | Fentanyl | 15 | 14 (93.3) | [68.1, 99.8] |  |  |  |  |
|  | Post-operation | Remifentanil | 82 | 82 (100.0) | [95.6, 100.0] |  | 1.3 | [-1.3, 3.9] | <0.0001 |
|  |  | Fentanyl | 75 | 74 (98.7) | [92.8, 100.0] |  |  |  |  |
| Concomitant sedatives | Propofol | Remifentanil | 50 | 50 (100.0) | [92.9, 100.0] |  | 4.3 | [-1.5, 10.0] | <0.0001 |
|  |  | Fentanyl | 47 | 45 (95.7) | [85.5, 99.5] |  |  |  |  |
|  | Dexmedetomidine | Remifentanil | 64 | 64 (100.0) | [94.4, 100.0] |  | 2.9 | [-1.1, 6.9] | <0.0001 |
|  |  | Fentanyl | 69 | 67 (97.1) | [89.9, 99.6] |  |  |  |  |

*CI* Confidence interval, *ICU* intensive care unit
